# Pathogenesis-Related Protein 1b1 (PR1b1) Is a Major Tomato Fruit Protein Responsive to Chilling Temperature and Upregulated in High Polyamine Transgenic Genotypes

**DOI:** 10.3389/fpls.2016.00901

**Published:** 2016-06-22

**Authors:** Ravinder K. Goyal, Tahira Fatima, Muhamet Topuz, Anne Bernadec, Richard Sicher, Avtar K. Handa, Autar K. Mattoo

**Affiliations:** ^1^Sustainable Agricultural Systems Laboratory, Agricultural Research Service, United States Department of Agriculture, BeltsvilleMD, USA; ^2^Crop Systems and Global Change Laboratory, The Henry A. Wallace Beltsville Agricultural Research Center, Agricultural Research Service – United States Department of Agriculture, BeltsvilleMD, USA; ^3^Department of Horticulture and Landscape Architecture, Purdue University, W. LafayetteIN, USA

**Keywords:** low temperature response, *Solanum lycopersicum*, cold responsive genes, methyl jasmonate, PR proteins, genetic engineering, 1-MCP, ethylene

## Abstract

Plants execute an array of mechanisms in response to stress which include upregulation of defense-related proteins and changes in specific metabolites. Polyamines – putrescine (Put), spermidine (Spd), and spermine (Spm) – are metabolites commonly found associated with abiotic stresses such as chilling stress. We have generated two transgenic tomato lines (556HO and 579HO) that express yeast *S*-adenosylmethionine decarboxylase and specifically accumulate Spd and Spm in fruits in comparison to fruits from control (556AZ) plants (Mehta et al., 2002). Tomato fruits undergo chilling injury at temperatures below 13°C. The high Spd and Spm tomato together with the control azygous line were utilized to address role(s) of polyamines in chilling-injury signaling. Exposure to chilling temperature (2°C) led to several-fold increase in the Put content in all the lines. Upon re-warming of the fruits at 20°C, the levels of Spd and Spm increased further in the fruit of the two transgenic lines, the higher levels remaining stable for 15 days after re-warming as compared to the fruit from the control line. Profiling their steady state proteins before and after re-warming highlighted a protein of ∼14 kD. Using proteomics approach, protein sequencing and immunoblotting, the ∼14-kD protein was identified as the pathogenesis related protein 1b1 (PR1b1). The PR1b1 protein accumulated transiently in the control fruit whose level was barely detectable at d 15 post-warming while in the fruit from both the 556HO and 579HO transgenic lines PR1b1 abundance increased and remained stable till d 15 post warming. *PR1b1* gene transcripts were found low in the control fruit with a visible accumulation only on d 15 post warming; however, in both the transgenic lines it accumulated and increased soon after rewarming being several-fold higher on day 2 while in 556HO line this increase continued until d 6 than the control fruit. The chilling-induced increase in PR1b1 protein seems independent of ethylene and methyl jasmonate signaling but may be linked to salicylic acid. We propose that polyamine-mediated sustained accumulation of PR1b1 protein in post-warmed chilled tomato fruit is a pre-emptive cold stress response and possibly a defense response mechanism related to Cold Stress-Induced Disease Resistance (SIDR) phenomenon.

## Introduction

Plants have evolved intricate mechanisms to survive vagaries of nature but some do better than others in mitigating detrimental effects upon exposure to extreme environmental stresses, including temperature extremes. At low temperatures, growth, quality and productivity of plants can be compromised. Temperatures below 0^0^C lead to freezing injury in major crops while temperatures above freezing point may cause physiological disorders such as chilling injury in diverse crops such as beans, corn, fruits, vegetables, and floriculture (Wang, 1989). In fruits, the chilling injury is manifested in the form of lesions, deterioration in quality, or even losing the ability to ripen normally and to produce viable seeds (Zhang et al., 2008; Farneti et al., 2015). The susceptibility of fruits to chilling injury not only limits their shelf life but also causes substantial economic losses. Low temperature tolerance is a complex, quantitative trait with molecular mechanisms yet to be fully understood.

Plant genotypes with low-temperature tolerance have a greater ability to maintain and/or repair membrane damage with reduced electrolyte leakage over the sensitive genotypes (Campos et al., 2003; Aroca et al., 2005). Increased lipid degradation or lateral phase separation in membranes at chilling temperatures render them leaky (Parkin and Kuo, 1989; Sharom et al., 1994), which gets intensified after return to ambient temperatures. Membrane structure and function at low temperature has been related to desaturation index of fatty acids (Murata and Los, 1997), and plants increase unsaturated fatty acid content in membrane lipids in response to freezing temperatures (Miquel et al., 1993; Chen and Thelen, 2013). Genetic engineering approach confirmed that enhancing unsaturated index of plant lipids confers chilling tolerance in some plants (Murata et al., 1992; Ishizaki-Nishizawa et al., 1996; Sui et al., 2007). Another aspect to chilling-mediated membrane damage involves oxidative damage by hydrogen peroxide (Prasad et al., 1994). Lignification is yet another defensive response against chilling injury in fruits (Vance et al., 1980; Zeng et al., 2015). Similarly, plants accumulate specific metabolites and proteins that can contribute to membrane stability against low temperature stress (Chen and Murata, 2002; Groppa and Benavides, 2008; Zhang et al., 2010).

Molecular approaches have identified a composite defense response of plants in response to chilling or cold temperatures, involving a plethora of transcription factors (Thomashow, 2010; Medina et al., 2011; Knight and Knight, 2012). Namely, the regulatory molecular cascade includes cold responsive genes/transcription factors ICE1 (Inducer of *CBF3* Expression1), MYB, MYC, and CBF, along with ubiquitin E3 ligase HOS1 and SUMO E3 ligases SIZ1/SIZ2. In *Arabidopsis, ICE1* encodes a MYC-like basic helix-loop-helix transcription factor that activates *CBF3/DREB1A* and *COR* genes in response to low temperatures (Chinnusamy et al., 2003). HOS1 acts as a negative regulator of *CBF3* expression through ubiquination of ICE1, which facilitates its degradation (Dong et al., 2006). Also, an interaction of *ICE1* and *MYB15*, and binding of the latter to promoters of CBF genes represses their expression and negatively regulates freezing tolerance (Agarwal et al., 2006). Similarly, *MYBs* have been associated with chilling symptomatology where some *MYB* members act as repressors or stimulators of lignin biosynthesis during chilling (Xu et al., 2014). SIZ1, on the other hand, stabilizes ICE1 by sumoylation and promotes freezing tolerance through positive regulation of *CBF3/DREB1A* expression (Miura et al., 2007). Also, ectopic expression of *ICE1* led to enhanced tolerance to cold stress which was concomitant with substantial increase in arginine decarboxylase (ADC) transcripts and levels of free polyamines (Huang et al., 2015).

Polyamines are a class of ubiquitous organic aliphatic cations constituting, among other minor components, diamine putrescine (Put), tri-amine spermidine (Spd), and tetra-amines spermine (Spm) and thermo-spermine (T-Spm), which have been implicated in protection against stresses such as chilling stress. Early research on plant responses to abiotic stress found increased accumulation of polyamines in response to osmotic shock (Flores and Galston, 1982), cold hardiness (Nadeau et al., 1987) and chilling injury (McDonald and Kushad, 1986). It is now commonly observed that polyamines prominently feature during plant responses to environmental stresses, including drought, salinity and extreme temperatures (Pang et al., 2007; Groppa and Benavides, 2008; Takahashi and Kakehi, 2010; Mattoo et al., 2015). Higher endogenous level of polyamines in response to chilling has been correlated with chilling tolerance in plants (Shen et al., 2000 and references therein). Conversely, reducing the accumulation of polyamines by inhibiting S-adenosylmethionine decarboxylase activity enhanced photoinhibition in spinach leaves at low temperature (He et al., 2002). Also, pre-treatment of chilling-sensitive zucchini fruit with Spd (Martínez-Téllez et al., 2002) or enhancing endogenous accumulation of *Spd* through overexpression of Spd synthase in *Arabidopsis* (Kasukabe et al., 2004) improved their chilling tolerance. Alleviation of chilling injury in cold-sensitive cucumber plants by exogenous Spd correlated with reduction in H_2_O_2_ content (Shen et al., 2000). Spd and Spm are known to stabilize cellular membranes against free radical-mediated lipid peroxidation (Tadolini et al., 1984; Larher et al., 2003). Spermine is known to freeze the increase in microviscosity of apple microsomal membranes at a constant value above 4–12°C (Ben-Arie et al., 1982).

From the foregoing it is apparent that polyamines likely form a part of composite plant response to abiotic stresses, including chilling temperatures. However, our understanding of their precise role in the defense processes in plants is still in infancy. To study chilling-injury signaling in regard to the involvement of Spd and Spm in tomato fruit, we utilized two transgenic tomato lines, 556HO and 579HO, that have been previously characterized (Mehta et al., 2002) and fruits of which accumulate higher polyamines, Spd and Spm, in comparison with a control line (556AZ). We demonstrate here that tomato fruit exposed to chilling temperature and re-warmed thereafter leads to a marked increase in polyamines, the levels of which are sustained for 15 days in the fruit from the two transgenic lines but transiently in the control 556AZ line. Further, using a proteomics approach we identify a ∼14 kD protein that characteristically accumulates in concert with the accumulation of Spd and Spm in 556HO and 579HO fruit and has a longer half-life than in the 556AZ line fruit. Protein sequence analysis and immunological evidence identified this protein as the pathogenesis-related 1b1 (PR1b1) protein. Finally, we show a positive correlation between increase in PR1b1 protein and gene transcripts, with those of *MYC2, MYB1, CBF1* and glucose-6-phosphate dehydrogenase (G-6-P DH) transcripts, and salicylic acid (SA) levels in the transgenic tomato fruit that accumulate Spd/Spm.

## Materials and Methods

### Plant Materials and Chilling Treatment

Tomato (*Solanum lycopersicum* Mill.) cultivar used as control and for developing transgenic lines was Ohio 8245. Transgenic tomato lines, 556HO and 579HO, homozygous for yeast S-adenosylmethionine decarboxylase (ySAMdc), its parent azygous line (556AZ) (Mehta et al., 2002; Neelam et al., 2008) and methyl jasmonate-deficient line (lox^-^) (Kausch et al., 2012) have been described. The different genotypes were grown in a greenhouse under natural light. To study the effects of chilling, first experiment involved exposure of mature green, breaker and pink tomato fruits from the control line at 4°C and 15°C. Then, at different days (see Supplementary Figure S1), samples were harvested and frozen at -80°C until used. Subsequently, for the remaining experiments, mature green fruits from the azygous line (556AZ; Mehta et al., 2002) and transgenic lines (556HO and 579HO) were exposed to 2°C for 14 days and then re-warmed at 20°C. Samples were taken as mentioned for up to 15 days. As a temperature control (non-chilled samples), mature green fruits from the latter three lines were also kept at 20°C and samples were removed on different days for analysis.

### Polyamine Analysis

Putrescine, spermidine, and spermine levels were analyzed from fruit pericarp by incubating 500 mg/tissue in 1 ml 5% perchloric acid and the samples were frozen at -20°C. Freezing and thawing of thus treated samples allowed solubilization of polyamines, insoluble residue was centrifuged down at 12,000*g* for 15 min, and the supernatants saved. Heptanediamine was added to the supernatants as an internal standard, the samples were dansylated and quantified as described previously (Minocha et al., 1990).

### Salicylic Acid Analysis

Freeze-dried tomato samples (50 mg each) were twice extracted with 1.5 ml of 70% MeOH in ground glass homogenizer at 4°C and supernatants were combined. Thereafter the methodology described previously was followed (Meuwly and Métraux, 1993). SA was separated using Waters HPLC model 600E Multisolvent Delivery System (5 μm, Luna RP-C18 column from Phenomenex, 150 mm × 4.4 mm) and detected by fluorescence (305 ex and 407 em) on Shimadzu RF-535 Spectrofluorimeter following the same method (Meuwly and Métraux, 1993). For internal standard, randomly a couple of samples extracted with 1.5 ml 70% MeOH were spiked with 40 μl of 1 mM SA standard.

### Protein Extraction

Frozen tomato fruit pericarp was powdered in a mortar with a pestle on ice and then extracted with a medium containing 50 mM Tris-Cl (1:1 w/v), pH 7.4, 10% (v/v) glycerol, 50 mM NaCl, 20 mM MgSO_4_, 1 mM EDTA, 5 mM 2-mercaptoethanol, 0.5 mM phenylmethylsulfonylfluoride, 10 μM leupeptin, and pepstatin A (1 μg^-1^ml) as described previously (Mehta et al., 1991). During extraction, insoluble polyvinylpolypyrrolidone (20 mg^-1^g FW) was also added and mixed thoroughly with the slurry. The homogenate was filtered through two layers of cheesecloth and then Mira cloth in a cold room. The filtrate was centrifuged at 25,000*g* for 30 min and supernatant was saved and used as soluble proteins.

### SDS-Polyacrylamide Gel Electrophoresis and Immunobloting

Proteins were solubilized in sample application buffer (Mattoo et al., 1981) and separated on 12% SDS-polyacrylamide Tris-glycine and 16.5% Tris-tricine gels using Laemmli sample buffer. The samples were loaded on an equal protein basis. The gels were either stained with Coomassie-blue R-250 or were electrotransferred to nitrocellulose membranes (0.1 μ, Schleicher & Schuell, Germany) in 25 mM Tris base, 192 mM glycine, 20% methanol, and 0.02% SDS. The nitrocellulose membranes were washed in high salt tris (HST) [20 mM Tris, pH 7.5, 1 M NaCl, and 0.5% (v/v) Tween-20] for 5 min, and then blocked with 2% BSA for 2 h. Following three washes in HST each for 5 min, the membranes were incubated overnight at 4°C with anti-PR1abc antibody diluted (1:2000) in 25 mM Tris-Cl, pH 7.4, 150 mM NaCl, 1% BSA, and 0.5% (v/v) Tween-20 (the antibody was a gift from Prof. Robert Fluhr; Lotan et al., 1989). Unbound primary antibody was washed off by treatment with HST thrice, each for 5 min. After incubation with goat anti-rabbit IgG conjugated to alkaline phosphatase (1:2500) (KPL, Bethesda, MD, USA), 2 h at 25°C, and three washes in HST, enzyme activity was developed with nitrotetrazolium blue chloride (NBT) and 5-bromo-4-chloro-3-indolyl phosphate (BCIP) in alkaline medium.

### Protein Microsequencing

The protein band(s) running at ∼14 kD in SDS-PAGE was excised, re-electrophoresed on SDS-PAGE gel, eluted and re-electrophoresed on Tris-tricine gels as in the previous section. The ∼14 kD protein band was subjected to in-gel tryptic digestion, the tryptic fragments were purified on HPLC (Counterman et al., 2003), and outsourced for analysis by MALDI-TOF Mass Spectrometry and sequencing. Batch MSMS database searching employed search programs using ProteinProspector^1^.

### RNA Isolation and Quantitative PCR Analysis

Total RNA was isolated from freeze dried pericarp tissue using the RNeasy Plant Mini Kit (Qiagen). RNA quantification and quality were determined using a spectrophotometer and native agarose gel electrophoresis, respectively. First strand cDNA synthesis was performed using the SuperScript^®^ cDNA Synthesis Kit (Invitrogen) following the manufacturer’s instructions, in a final volume of 20 μl. The final cDNA products were diluted 10-fold prior to use in real-time (qPCR). The targeted cold responsive genes were selected on the basis of their known function in the literature and elements identified on tomato *PR1b1* gene at the 5′ end. Quantitative RT-PCR was performed with gene-specific primers (accession number and other information including specific allelic variants are given in Supplementary Table S1). The mRNA or complete coding sequences were used to design primers by using primers3 software, and the specificity was further confirmed by Primer–Blast^2^. The RT-PCR conditions were the same as before (Fatima et al., 2012). Tomato actin gene was used as reference for normalization. The relative expression levels of transcripts were quantified by the comparative CT method using the 2^-ΔΔCT^ formula using azygous (556AZ) fruit on day 0 of rewarming as a calibrator and the data are expressed as fold change.

### Statistical Analysis

**FIGURE 1:**
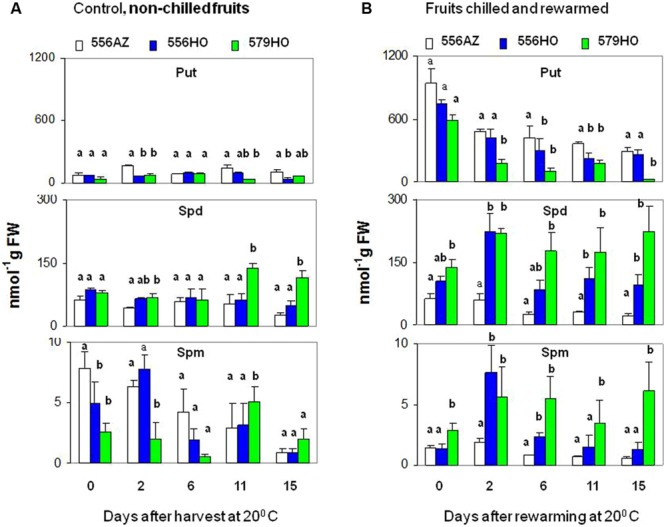
**Changes in the levels of putrescine (Put), spermidine (Spd), and spermine (Spm) in **(A)** tomato fruits harvested at mature green stage (indicated by day 0) from the indicated lines and held at 20°C, representing controls for samples shown in **(B)** for which mature green fruits harvested from the indicated lines were first chilled by incubation at 2°C for 14 days (day 0) and then re-warmed at 20°C for the indicated days.** Samples in triplicate were removed for each line at days 2, 6, 11, and 15, and analyzed for the content of each polyamine. Bars represent standard error of means (*n* = 3). Data sets were evaluated by one-way analysis of variance (ANOVA) with Tukey’s *post hoc* multiple comparison test to find differences among three lines for each polyamine. Single letters mark the top of each bar, those that share similar letters were found not significantly different at *P* < 0.05.

Statistical analysis of the changes in putrescine, spermidine, and spermine levels in tomato fruit given different treatments was performed with XLSTAT version 2016.1 (Microsoft Excel). Data are presented as the mean ± SEM of triplicates from three independent samples. Data sets were evaluated by one-way analysis of variance (ANOVA) to compare groups (corresponding to different lines at indicated times and temperature) with Tukey’s (HSD) *post hoc* multiple comparison test to determine statistical significance of differences between means within groups, *P* < 0.05 was considered as statistically significant (**Figure 1**). Any significant changes in the levels of SA between 566AZ and 579HO genotypes were determined for specified days after rewarming of chilled fruits using Student’s *t*-test (Microsoft Excel). ^∗^*P* < 0.08, ^∗∗^*P* < 0.05 (**Figure 4D**). Statistical analysis of Q-PCR quantified relative expression of indicated genes was also determined by Student’s *t*-test (Microsoft Excel), comparing all treatments to the values found for each gene in the control 566AZ genotype at day 0. Each bar represents the mean with standard error of three independent replications. *P*-values of significance represented by ^∗^(*P* < 0.05) and ^∗∗^(*P* < 0.01) (**Figures 5** and **6**).

## Results

### Diamine and Polyamine Profiles in Tomato Fruits under Two Contrasting Temperatures

Initial experiments were geared to monitor temperature effects on the levels of Put, Spd and Spm in tomato fruits (*Solanum lycopersicum* Mill, Ohio 8245 – control line) ripened on the vine and harvested at mature green, breaker and pink stages and then exposed to 4°C or 15°C for up to 20–30 days (Supplementary Figure S1). It was reasoned that ripening would be slow but normal at 15°C while the fruit held at 4°C would result in low temperature-induced changes, including effects on the levels of polyamines. In fact, Put levels in fruit harvested at breaker and pink stages were ∼50% of that in the green fruit, with about threefold higher levels of Put being apparent in the mature green fruit held at 4°C. In contrast, Spd and Spm levels in the mature green and breaker stage fruit held at 4°C initially decreased more than the fruit held at 15°C, but by 14 days the levels rose to those in the 15°C-held fruit (Supplementary Figure S1, Spd, Spm; MG versus BR). The patterns of changes in Put, Spd and Spm in the pink-stage fruit were more or less similar at both the temperature regimes (Supplementary Figure S1, PK). It would appear from these patterns that the green and breaker stage fruits held at 4°C respond at a slower pace in terms of polyamine metabolism than the fruit held at 15°C likely due to lower metabolic activity. This differential response, which lasted 14 days of incubation, was not apparent in the pink fruit. These observations are in line with previous observations showing more sensitivity of green fruit to chilling temperatures (Furlong, 1946).

### Chilling Temperature Response of Tomato Fruit Engineered for Higher Polyamine Accumulation

To further study implications of chilling temperature on the dynamics of polyamine metabolism, we selected mature green fruit from transgenic tomato lines (lines 556HO and 579HO, described previously in Mehta et al., 2002 and Neelam et al., 2008) genetically engineered for accumulation of higher polyamines, Spd and Spm, as well as the azygous (line 556AZ; Mehta et al., 2002) control. Fruits from the three lines were incubated at either 20°C (room temperature control) or 2°C (more severe chilling injury temperature than 4°C). Major and differential changes in the contents of Put, Spd, and Spm were apparent in the fruits held for 14 days at 2°C and then returned to 20°C (**Figure 1**). Put level increased by 10 to 12-fold in both transgenic and azygous fruits on day 14 of exposure at 2°C as compared to control fruit held at 20°C. Once fruits were returned to ambient temperature, Put level precipitously declined and the decrease progressed with time in all the three lines. In contrast to the dramatic change in Put content, the content of Spd on day 14 of exposure to chilling temperature was not as dramatically increased compared to the control fruit held at 20°C (**Figure 1**, Spd). However, following the temperature shift to 20°C after 14 days at 2°C Spd levels in both the transgenic lines (556HO and 579HO) increased sharply by day 2 which were significantly maintained at a higher level thereafter as compared to the azygous control line. The accumulation pattern for Spm content, which comparatively accumulated at low levels, was found similar to Spd in transgenic fruits (**Figure 1**, Spm). The difference in Spm content between the azygous and transgenic fruits was significant up to 6 days of re-warming (DAR) with the 579HO fruit maintaining a higher threshold level.

### Identification of Pathogenesis Responsive Protein PR1b1 as a Major Chilling-Induced Protein Whose Accumulation Is Sustained in Tomato Fruit that Accumulate Spd/Spm

**FIGURE 2:**
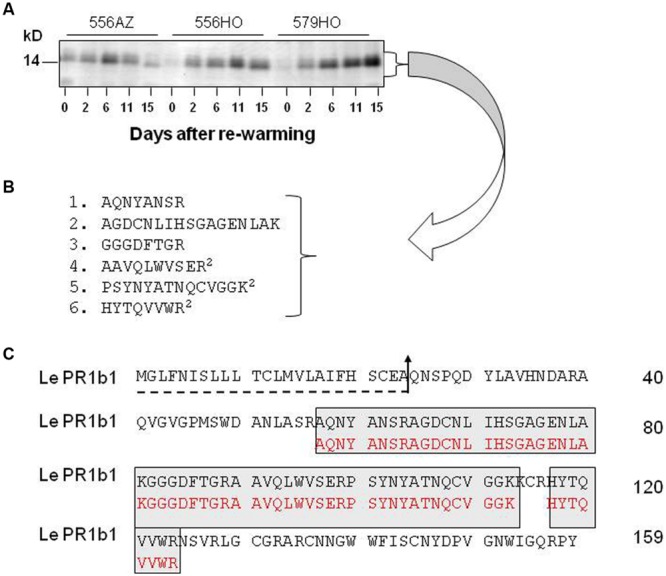
**The chilling-induced ∼14 kD protein is PR1b1. (A)** Shown is a Coomassie-blue R-250 stained SDS-PAGE gel (Supplementary Figure S1) strip around the ∼14 kD region following fractionation of total soluble protein from samples taken on the days shown after re-warming the chilled fruits at 20°C (14-d chilled fruit is designated by day 0). Each lane contained equal amount of protein. **(B)** Micro sequencing analysis of chilling-induced ∼14 kD protein. The protein was purified, eluted and trypsinized and amino acid sequence of tryptic-peptides was determined by MALDI-MS. The superscripts indicate the times those particular peptides were re-sequenced. **(C)** Alignment of the protein amino acid sequence (lower lanes in red) with deduced amino acid sequence of tomato PR1b1 (upper lanes). The dotted line with arrow shows the putative cleavage site of hydrophobic signal peptide.

To discern changes in steady state levels of soluble proteins, we compared total soluble protein profiles of control 556AZ and transgenic 556HO and 579HO fruits during normal ripening at 20°C as well as following re-warming after 14 days at 2°C. SDS-PAGE revealed differential accumulation of a number of proteins (Supplementary Figures S2A,B), but notable was a protein of ∼14 kD size whose content accumulated particularly and immediately during warming of the 14-day-chilled fruits (see Supplementary Figures S2A,B). The ∼14-kD protein accumulated in the fruit from all the three lines tested but with a different pattern: the protein accumulation in the 556AZ fruit peaked at 6 days after re-warming (DAR), declining thereafter to barely detectable levels by 15 DAR; in contrast, the ∼14 kD protein continued to accumulate during the 15-days’ re-warming period in both the transgenic fruits (556HO and 579HO) that accumulate Spd/Spm (**Figure 2A**).

For MALDI-MS protein sequencing, we eluted the ∼ 14-kD protein band(s) bulking it before partial purification and subsequent trypsinization. Micro-sequencing of the trypsin digests revealed six distinct amino acid fragmentation, three of which were duplicated (**Figure 2B**, sequences 4–6). A BLAST search of these amino acid sequences revealed the protein to be a member of pathogenesis-related (PR) proteins (**Figure 2C**); the protein sequence was identical to the deduced amino acid sequence of tomato PR1b1 (Tornero et al., 1997), designated previously also as P6 (van Kan et al., 1992). The six tryptic-peptides that were sequenced (>50%) were in congruence with trypsin cleavage sites (**Figures 2B,C**). The PR1b1 protein is 159 amino acids long with a putative 24-amino acid hydrophobic signal at the N-terminus (von Heijne, 1983; Lucas et al., 1985). The removal of signal peptide leaves 135 amino acids long protein with a calculated pI of 8.94 and molecular weight of 14.8 kD, the latter being close to that revealed by SDS-PAGE (**Figure 2A**). A possibility of other protein(s) co-migrating with PR1b1 is a distant one since all the six tryptic fragment sequences identically matched with the known PR1b1 protein sequence.

**FIGURE 3:**
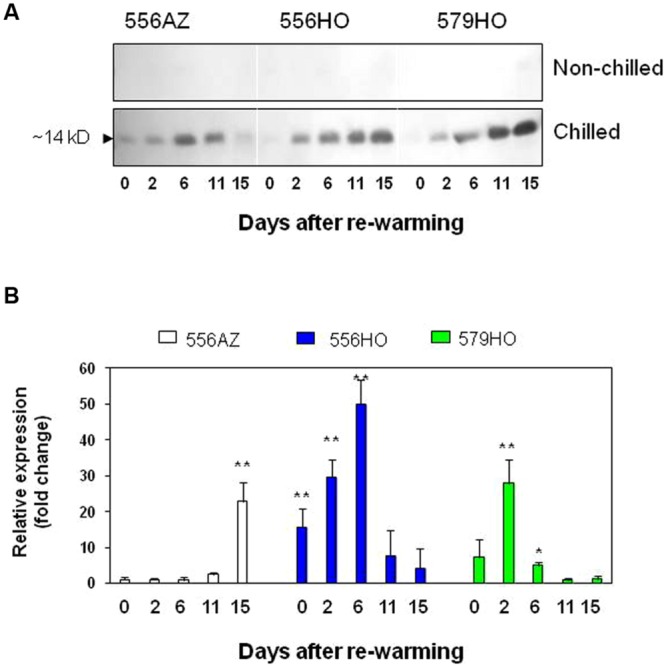
**Immunodetection of ∼14 kD protein and gene expression of PR1b1 gene.** Soluble proteins were isolated from both non-chilled and chilled fruits of azygous and transgenic lines, and Q-PCR analysis of PR1b1 transcripts was carried out RNA isolated from chilled samples following their transfer to rewarming temperatiure of 20°C. **(A)** Immunoblot analysis with anti-PR1abc antibody of soluble proteins. An arrow points to the immunodetected ∼14 kD protein. Other details were the same as described in the legend to **Figure 1**. **(B)** Q-PCR analysis of PR1b1 at different days after rewarming. The transcripts expression levels were determined relative to 0 day sample of the calibrator azygous (556AZ) line. Data set was analyzed by Student’s *t*-test (Microsoft Excel) comparing control 566AZ at 0 day with other treatments. Each bar represents the mean with standard error of three independent replications. P values are represented as ^∗^*P* < 0.05, ^∗∗^*P* < 0.01.

Immunoblot analysis using anti-PR1abc antibody further confirmed that the ∼14-kD tomato fruit protein belongs to a group of PR-proteins. The immunoblot results (**Figure 3A**) depicted a strong cross-reaction with this antibody and showed an identical pattern of accumulation to that observed on Coomassie-blue staining of the gel (**Figure 2A** and Supplementary Figure S2B). The chilling-induced PR1b1 protein was below detectable limits in the control fruits (**Figure 3A**, Non-chilled), indicating that its upregulation is related to the fruit’s exposure to chilling temperature, making it a major protein that accumulates upon chilling of tomato fruit.

### Polyamines Spd/Spm and Chilling Exposure Synergistically Up-Regulate PR1b1 Transcripts

To ascertain if PR1b1 transcripts accumulate in a pattern similar to that of the protein, we quantified the PR1b1 transcripts of fruits using real time PCR. The *PR1b1* from tomato (accession number Y08804) along with actin (ACT7) reference gene from tomato (accession number AB199316) were detected using SYBR green dye. The analysis of fruits showed that, like its protein, PR1b1 mRNA too is induced upon chilling, though with different timelines for the control versus transgenic lines (**Figure 3B**). In transgenic (556HO and 579HO) fruits, accumulation of the PR1b1 transcript levels was significantly increased at 2 and 6 DAR with the levels being significantly several-fold higher compared to azygous control in which *PR1b1* expression was insignificant until 15 DAR. However, PR1b1 transcripts accumulated ahead of its protein product in both the transgenic fruits whereas the transcripts in azygous control were maximally present on 15 DAR when the corresponding protein was barely detectable (compare **Figure 3B** with **Figure 3A**, 556AZ samples). Accumulation pattern of PR1b protein closely resembles that seen in potato leaves upon infection by *Phytophthora infestans* (Hoegen et al., 2002). Thus, higher polyamines and chilling stress have a synergistic positive effect on the PR1b1 transcript accumulation and that of its protein.

### Plant Hormones and Regulation of Chilling-Induced PR1b1 Accumulation in Tomato Fruit

Jasmonates, constituting jasmonic acid and its methyl ester, have been implicated in signal transduction during plant abiotic and biotic stress responses, particularly during wounding and pathogenesis (Farmer and Ryan, 1990; Li et al., 2002). It has also been shown that ethylene-dependent signal transduction pathway mediates the induction of basic isoforms of PR-1, including PR1b1, in response to pathogen attack (Sessa et al., 1995; Tornero et al., 1997). In addition to ethylene, jasmonates (meJA) and SA seem to regulate induction of PR-proteins (Ding et al., 2002; van Loon et al., 2006). Therefore, we examined if any or all of these three hormones are involved in the induction of PR1b1 protein in tomato fruit under chilling and re-warming conditions.

**FIGURE 4:**
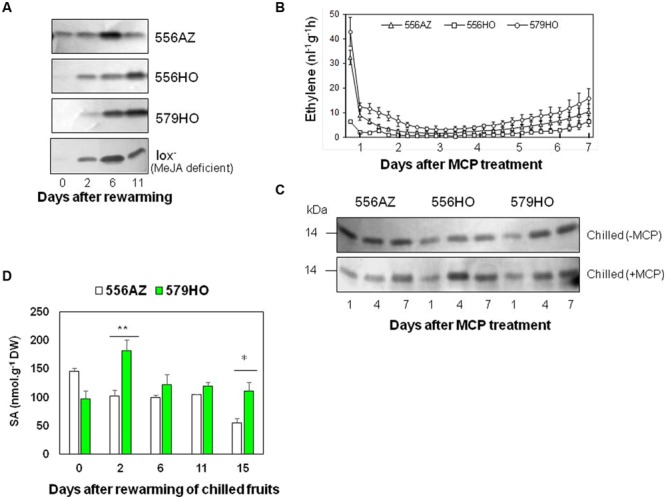
**(A)** Accumulation of PR1b1 protein in methyl jasmonate-deficient (lox^-^) fruit in comparison to azygous control (556AZ) and SAMdc transgenic (556HO, 579HO) fruits upon chilling and re-warming. **(B,C)** Effect of MCP treatment of fruits from the different lines on ethylene production **(B)**, and PR1b1 protein accumulation **(C)**. The fruits, immediately after chilling were treated with 5 ppm MCP for 24 h at 20°C and analyzed for: **(B)** Rate of ethylene production measured at intervals during the course of 7 days (bars represent standard error of means; *n* = 4); **(C)** accumulation of PR1b1 protein in MCP-treated (+MCP) and control (–MCP) fruits. PR1b1 accumulation was determined by immunoblotting using anti PR1abc antibody. **(D)** Salicylic acid (SA) levels in azygous control (556AZ) and SAMdc transgenic (579HO) fruits. The fruits were chilled and re-warmed as described in the legend to **Figure 1**. Statistically significant values of SA between 566AZ and 579HO at the specified days after rewarming of chilled fruits are marked on the bars with ^∗^ (*P* < 0.08) and ^∗∗^ (*P* < 0.05).

Involvement of meJA in chilling-induced PR1b1 accumulation was studied using the meJA-deficient, mutant tomato fruit (Kausch et al., 2012). Chilling of these meJA-deficient fruit followed by re-warming resulted in the accumulation of PR1b1, following an identical pattern as the control 556AZ tomato fruit (**Figure 4A**). Thus, meJA deficiency does not limit the induction of PR1b1 upon chilling. These data are in agreement with inability of JA to induce PR-1b mRNA in potato (Hoegen et al., 2002).

For the involvement of ethylene in the induction of PR1b1, tomato fruits (556AZ, 556HO, and 579HO) were chilled for 14 days as mentioned above and before their re-warming they were incubated at 2°C in an air-tight desiccator with 5 ppm 1-methylcyclopropene (MCP), an ethylene action blocker (Blankenship and Dole, 2003). MCP treatment led to a drastic and immediate reduction in ethylene production to near zero value between 3 and 4 DAR before exhibiting an increase (**Figure 4B**). Under these conditions, however, the PR1b1 protein induction remained unaffected in both the azygous and transgenic fruits (**Figure 4C**).

The involvement of the third signaling hormone, SA, was studied by quantifying its content in previously chilled 556AZ and 579HO fruits upon re-warming. Immediately after re-warming, the transgenic fruit had substantially higher SA content starting 2 DAR than the control fruit, with the SA content remaining higher in 579HO fruit on 15 DAR compared to the control fruit (**Figure 4D**). These data indicate that SA signaling may be a factor in the induction of PR1b1 protein upon chilling. This is interesting because SA is known to be synthesized by plants in response to pathogen attack and upregulates PR protein genes (Ding et al., 2002; van Loon et al., 2006).

### PR1b1 Gene Promoter Harbors MYB and MYC Elements Which Are Chilling Upregulated

**FIGURE 5:**
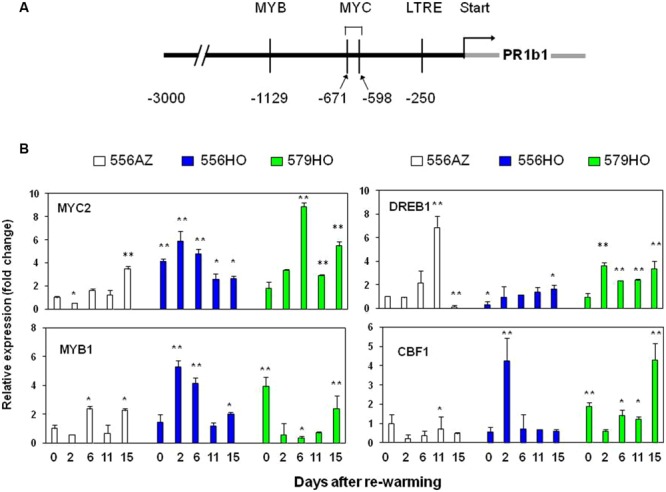
**(A)** Promoter analysis of tomato PR1b1 gene. Up to 3 Kb of the promoter was analyzed by using online PLACE (http://www.dna.affrc.go.jp/PLACE/) tool. The forward arrow indicates the beginning of ORF. LTRE: Low temperature responsive element. **(B)** Quantitative PCR (Q-PCR) analysis of MYB1, MYC2, DREB1, and CBF1 transcripts was performed on RNA isolated from tomato fruit pericarp tissue sampled at 0 (chilled sample, 14-d at 2°C), 2, 6, 11, and 15 days after re-warming (DAR) at 20°C as described in “Materials and Methods”. The relative expression levels of transcripts were quantified by using the 2^-ΔΔCT^ method using azygous (556AZ) at 0 day as the calibrator and expressed as fold change. For statistical analyses, transcripts of each gene on different days were compared with that in the control 566AZ line on day 0. Each bar represents the mean with standard error of three independent replications. Relative expression data were analyzed by Student’s *t*-test, ^∗^*P* < 0.05 and ^∗∗^*P* < 0.01 (**Figures 5** and **6**).

Since chilling temperatures induced PR1b1 transcripts and its protein, we surmised that its gene promoter may harbor cold responsive elements. Therefore, we used online tool PLACE^3^ and performed promoter analysis of 3 Kb region 5′ of tomato PR1b1 gene. Results of this analysis revealed the presence of low temperature responsive element (LTRE) (at -250), two MYC elements (at -598 and -671) and MYB element (at -1129) at the indicated regions 5′ of tomato PR1b1 gene (**Figure 5A**). We did not find the CBF *cis* element (RYCGAC), which is part of CBF/DREB1-dependent cold signaling pathway in plants, in the promoter region of the tomato PR1b1 gene (Miura and Furumoto, 2013 and references therein).

Next, we quantified by Q-PCR the patterns of accumulation of *MYC2* and *MYB1* transcription factors that were found resident at the 5′ promoter region of tomato PR1b1 gene (**Figure 5A**) as well as of the tomato *DREB1* and *CBF1* transcription factors in relation to the chilling and re-warming conditions used for studying PR1b1 expression as described above (**Figure 5B**). During the re-warming of the chilling-exposed tomato fruit from the three lines, the quantified transcription factors were found up-regulated.

Overall, MYC2 transcripts were upregulated in both the high polyamine transgenic 556HO and 579HO lines (elevated in Spd/Spm content), with maximum transcript levels seen at 2-6 DAR than the 556AZ control fruit in which *MYC2* transcript levels were generally low, being highest at 15 DAR (**Figure 5B**, panel MYC2). The expression profile of *MYB1* was more or less similar to that of *MYC2* except that it largely accumulated in the 579HO fruit particularly during chilling (see the 0 DAR sample). *DREB1* expression was highest in the control 556AZ line at 11 DAR, precipitously decreasing to almost undetectable levels by 15 DAR (**Figure 5B**, DREB1). Relatively, DREB1 levels increased in both the transgenic lines by 2 DAR and these levels were maintained all through to 15 DAR (**Figure 5B**, DREB1).

The transcript level of *CBF1* remained steadily low in the fruits of azygous control and the 556HO transgenic line throughout the re-warming period (**Figure 5B**, CBF1). However, substantial up-regulation of *CBF1* at 0 and 15 DAR was found in 579HO line, the pattern mirroring that seen for MYB1 in this line (**Figure 5**, compare *MYB1* and *CBF1*).

### Marker Genes of Nitrogen (N) and Carbon (C) Metabolism Display Inverse Expression in Control versus Transgenic Fruits upon Re-Warming after Chilling

**FIGURE 6:**
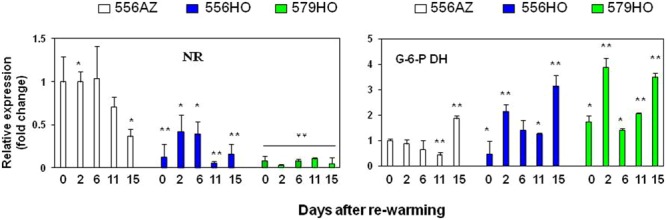
**Quantitative PCR (Q-PCR) analysis of nitrate reductase (NR) and glucose-6-phosphate dehydrogenase (G-6-P DH) transcripts in tomato fruit pericarp samples.** Methods including data quantification and statistical analysis were the same as described in the legend to **Figure 5**.

Polyamines have been implicated in regulating N:C metabolism of the reproductive organs, including tomato fruit (Handa and Mattoo, 2010; Moschou et al., 2012). We therefore quantified transcript levels of nitrate reductase (*NR*) and glucose-6-phosphate dehydrogenase (*G-6-P DH*), two key genes that regulate, respectively, N and C metabolism. The fruit from the control 556AZ line had substantial abundance and higher *NR* expression at 0, 2, and 6 DAR decreasing thereafter to low levels at 15 DAR (**Figure 6**, NR). In contrast, NR expression was less abundant and remained significantly low in both the transgenic, high polyamine lines during the re-warming period (**Figure 6**, NR, 556HO, and 579HO), suggesting that higher polyamines suppress the activation of *NR* expression during chilling as well as upon rewarming. On the other hand, transcript levels of the C metabolism marker – *G-6-P DH*, were significantly higher in both the transgenic lines throughout the rewarming period as compared to the control azygous line (**Figure 6**, G-6-P DH).

## Discussion

Many plants, including wheat, soybeans, avocados, bananas, and Solanaceae family (e.g., eggplant, potato, tomato), suffer from chilling injury when exposed to temperatures below 8–15°C, subsequently affecting their development, ripening and pathogen response, and eventually their marketability (Lyons, 1973; Nishida and Murata, 1996; Lukatkin et al., 2012). Plant response to chilling temperatures above freezing involve complex processes, including alterations in physiology, metabolism and macromolecular processes dependent upon the crop, the temperature and time of exposure (see review by Miura and Furumoto, 2013). Polyamines prominently feature among the metabolites that accumulate in plants exposed to cold temperatures, but their precise signaling process still remains to be elucidated (Alcázar et al., 2011; Mattoo et al., 2015). We show here that exposure of tomato fruit for 14 days to 2°C (and re-warmed thereafter) leads to a marked increase in polyamines, transiently in the control 556AZ line but particularly sustained for 15 days (after re-warming to 20°C) in the fruit from two transgenic tomato lines engineered for the accumulation of higher polyamines, Spd and Spm. Further, we present proteomics-type approach, protein sequencing and immunological evidence to identify a ∼14 kD protein as the PR protein PR1b1 that characteristically accumulates in concert with the accumulation of Spd and Spm in the transgenic fruit (556HO and 579HO lines). Abundance of PR1b1 protein was preceded by a significant enhancement in *PR1b1* gene expression. *In silico* analysis showed that *MYB1* and *MYC2* transcriptional factors decorate 5′ end of tomato *PR1b1* gene promoter. Interestingly, PR1b1 accumulation was chilling specific since PR1b1 protein was not detected in the control, non-chilled fruit. The PR1b1 protein induction profile was found independent of the presence or absence of plant hormones ethylene and methyl jasmonate, but correlated with SA content, expression patterns of *MYC2, MYB1* and *CBF1* transcripts, and accumulation of polyamines Spd and Spm.

Accumulation of PR1b1 protein was concomitant with increased and sustained levels of Spd, Spm throughout the 15 days of re-warming period of the transgenic fruits while *PR1b1* transcripts massively accumulated on day 2 of re-warming. Moreover, expression of *MYB1* and *MYC2* transcription factors was sustained and paralleled the PR1b1 protein accumulation in the high Spd/Spm transgenic lines, demonstrating functional relevance of MYB and MYC elements in *PR1b1* promoter. Upregulation of the latter elements at cooler temperatures is known (e.g., Tran et al., 2004; Franco-Zorrilla et al., 2014; Xu et al., 2014). The chilling-mediated induction of MYB1/MYC2 transcription factors may be positive regulators of Put synthesis since arginine decarboxylase (ADC) activity was reported to increase upon chilling (Hao et al., 2005); however, its relevance to this study remains to be demonstrated. Also, *ICE1*, a MYC-like basic helix-loop-helix transcription factor, was reported to interact with *ADC* gene as was also found for *MYB* resulting in an increase of polyamine levels (Sun et al., 2014; Huang et al., 2015). It is possible but not yet defined that *MYB1* and *MYC2* upregulation could similarly interact with the tomato polyamine pathway, becoming an additional factor in furthering the enhancement of Spd and Spm levels in the transgenic tomato lines, harboring highly active yeast SAM decarboxylase transgene, and stabilize the levels of PR1b1 protein. Clearly, more experimental evidence is needed to support this hypothesis. Another possible scenario is that induction of polyamines at chilling temperatures could serve at least two purposes: One, to provide osmotolerance/protection to fruit membranes against oxidative stress (Ben-Arie et al., 1982; Souzu, 1986; Ha et al., 1998; Groppa et al., 2001) and second, to induce and stabilize PR1b1 protein. Spd and Spm are known to modulate protein synthesis by inducing structural changes in RNA structure (Igarashi and Kashiwagi, 2000) and stabilize proteins against degradation (Gugliucci and Menini, 2003). It seems likely that Spd/Spm stabilize and help sustain PR1b1 protein in the transgenic lines but not so much in the control line because, in fact, the levels of Spd/Spm in the control line are limiting on days 6–15 after re-warming.

Polyamines, especially Put, and osmolytes, including proline, sugars, glycine betaine, and sorbitol accumulate in response to abiotic stresses (Zhang et al., 1999; Montesinos-Pereira et al., 2014; Mattoo et al., 2015). Due to their positive charge, polyamines bind negatively charged residues in membranes and thereby influence membrane function (Schuber, 1989). They also facilitate recovery of both H^+^/ATPase and Ca^2+^/ATPase transporters from stress-damage (Roy et al., 2005), as well as detoxification of reactive oxygen species (ROS) to maintain ROS homoeostasis during stress (Wang et al., 2011; Saha et al., 2015). Fruits given a heat treatment accumulate polyamines and when these fruits are subsequently transferred to chilling temperature, the fruits seem protected against chilling (Zhang et al., 2013). Further, Put mutants display reduced tolerance to freezing temperatures (Cuevas et al., 2008), whereas Spd increase during re-warming of chilling-tolerant plants or pre-treatment of sensitive cultivars with Spd prevented their chilling injury (Shen et al., 2000). Similarly, transgenic plants that accumulated Spm were found better protected against chilling injury and other stresses (Kasukabe et al., 2004). These studies suggested that Put accumulation and its conversion to higher polyamines is a part of plant mechanism to mitigate the chilling stress. However, our studies reported here were not geared to study tolerance potential of increased Spd/Spm against chilling temperature but rather to demonstrate that a PR protein may be part of a signaling mechanism in response to chilling temperatures (also see below).

We have previously demonstrated that upregulation of Spd and Spm modulates specific cellular metabolism under ambient conditions (Mattoo et al., 2006). Here we show that exposure to chilling temperature followed by re-warming results in a shift in C and N metabolism, with C metabolism (*G-6-P DH* transcript abundance) predominant in the transgenic, high Spd/Spm fruits while N metabolism (*NR* transcript abundance) being predominant in the low Spd/Spm azygous fruits. The differential C and N metabolism could be an important response in plants to modulate cellular metabolism under unfavorable temperature conditions.

Pathogenesis-related proteins comprise a large family of plant proteins that are developmentally regulated (Lotan et al., 1989) and induced in response to pathogen infestation and abiotic stresses, *PR-1* homologs featuring prominently among them (Ernst et al., 1992; Green and Fluhr, 1995; Jakab et al., 2005). Plant hormones such as ethylene, SA, and jasmonic acid affect PR gene expression during different stresses (Yang et al., 1997; Reymond and Farmer, 1998; van Loon et al., 2006). However, PR proteins do not seem obligatory for PR-mediated resistance against pathogens (Smart et al., 2003). In the present study, we found that neither methyl jasmonate nor ethylene are necessary for chilling-induced PR1b1 protein accumulation. However, it was apparent that sustained accumulation of the PR1b1 protein in the high-polyamine lines correlated well with significantly higher level of SA on d 2 and 15 after rewarming of the fruits (**Figure 4D**). Although these findings are preliminary in nature, these studies seem in line with other studies showing alleviation of chilling injury by SA pre-treatment in tomato fruit, suggesting a role for this hormone in the chilling tolerance mechanism (Aghdam et al., 2014). In this regard, our data are consistent with the previous suggestion that spermine and SA act as signaling molecules for the accumulation of acidic PR-1 protein during TMV infection of tobacco leaves (Yamakawa et al., 1998).

The role of PR-1 class of PR proteins in chilling tolerance and/or host-derived protection against pathogens is not fully understood. Research on homologous PR-1 proteins show them to be developmentally regulated in *Arabidopsis*, rice and tobacco, and it has been suggested that in specific organs or tissues they may serve functions, other than plant defense (van Loon et al., 2006). Notably in this regard, PR proteins other than PR-1 class are commonly found associated with cold acclimation or stress of plants and it was hypothesized that they could be involved as components of stress-regulated signal transduction pathway (Renaut et al., 2006; Gharechahi et al., 2014). Over expression of PR1b in tobacco did not impart resistance against viruses (Cutt et al., 1989), although three basic homologous but distinct 14-kD proteins – P14a, P14b, P14c -, were found to possess antifungal activity (Niderman et al., 1995).

Pathogenesis-related 1b1 protein function has remained elusive. However, it may play a role as an anti-cold/anti-freeze protein since some antifreeze proteins are similar to some members of PR-proteins in cold acclimated rye (Hon et al., 1995). Finally, in addition to a role in chilling stress response signaling pathway, polyamine-mediated sustained accumulation of PR1b1 protein in tomato fruit during re-warming after exposure to chilling temperatures could be a pre-emptive plant defense mechanism like the previously described Cold Stress-Induced Disease Resistance (SIDR) phenomenon, genetic mechanisms of which have yet to be elucidated (Moyer et al., 2016). The latter cold SIDR phenomenon developed from the observation that low temperature exposure of sensitive plants made them less susceptible to pathogen infection by reducing infection success rates and slowing colonization (Moyer et al., 2010, 2016). In this context, the accumulation of PR1b1 protein in tomato upon chilling followed by re-warming might be a cold SIDR-like response, and since cold initiates polyamine accumulation prior to re-warming, it is tempting to speculate that polyamines prime the fruits to pre-empt any possible pathogen infection by activating *PR1b1* gene and accumulation of its protein product. Inevitably, longer and steady accumulation of Spd/Spm in the two transgenic tomato fruit could further stabilize and maintain longer half life of PR1b1 protein. The high Spd/Spm tomato fruit lines could be a good model for further studies to discern the genetic mechanism(s) of cold SIDR, signaling function of Spd/Spm, and the role of PR1b1 in this phenomenon.

## Author Contributions

RG and TF equally contributed to this manuscript; RG, TF, and AM conceived and designed the study; RG, TF, MT, AB, and RS participated in experimentation; AH provided reagents and edited the manuscript; TF contributed to writing parts of the manuscript; RG and AM wrote the manuscript.

## Conflict of Interest Statement

The authors declare that the research was conducted in the absence of any commercial or financial relationships that could be construed as a potential conflict of interest.

## Footnotes

1 http://prospector.ucsf.edu

2 www.ncbi.nlm.nih.gov/tools/primer-blast/

3 http://www.dna.affrc.go.jp/PLACE/
