# Supplementary material for: Pathogenesis-Related Protein 1b1 (PR1b1) Is a Major Tomato Fruit Protein Responsive to Chilling Temperature and Upregulated in High Polyamine Transgenic Genotypes
Source: Front Plant Sci. 2016 Jun 22;7:901. doi: 10.3389/fpls.2016.00901 (PMC4916175; doi:10.3389/fpls.2016.00901)

Supplementary Table 1. Tomato genes, their accession numbers and the primer sequence combinations used in Q-PCR analysis

**Gene/symbol**	**Accession and version /Source**	**Forward (5'-3')**	**Reverse (5'-3')**
Actin-7 (ACT)	AB199316.1/Tomato	ATGCGTATGTGGGTGATGAA	GCCTCAGTCAGGAGAACAGG
Pathogenesis-related protein (PR1B1)	Y08804.1/Tomato	TTGTGAGGCCCAAAATTCA	AGATTCTCCCCAGCACCAG
MYC transcription factor (jamyc2)	AJ630505.1/Potato	TGAATACAGCTTACCCACCA	CCAACCACAGCAGCAGAA
MYB transcription factor (MYB-1)	AY131230.1/Tomato	TTGAGATGCGGAAAGAGTTG	TTCATTATCTGTTCGTCCTGGT
Dehydration responsive element binding protein (DREB1)	AF500011.1/Tomato	TTTGTGTGCCGTTGGATT	ATTTTCTGGTCCCCCTTTAC
Putative transcriptional activator (CBF1)	AY034473.1/Tomato	AGGGGAATCAGGAAGAGGAA	GCCTCCAAGCAGAATCAGAG
Nitrate reductase (NR, nia)	X14060/Tomato	TGAAGACGAGGATGACGATG	GTTCGGAGTTGAATGGGTGT
Glucose-6-phosphate 1-dehydrogenase (G6PD)	AK246881.1/Tomato	GCAGGATTGATAAGAGGGAAAA	TTGGTGGAATGGACAAGTAGAA

Supplementary Fig. 1. Changes in Put, Spd and Spm levels in control tomato fruit (Ohio 8245 cv) during ripening when held at 4 ^{0}C or 15 ^{0}C for the indicated days. MG, mature green; BR, breaker; PK, pink. L1T4 and L1T15 represent incubation temperatures 4 ^{0}C and 15 ^{0}C, respectively. Fruits were transferred from 4 ^{0}C and 15 ^{0}C to 20 ^{0}C on day 14, as indicated by the arrow in the top panel. Bars represent standard error of means (n = 3).

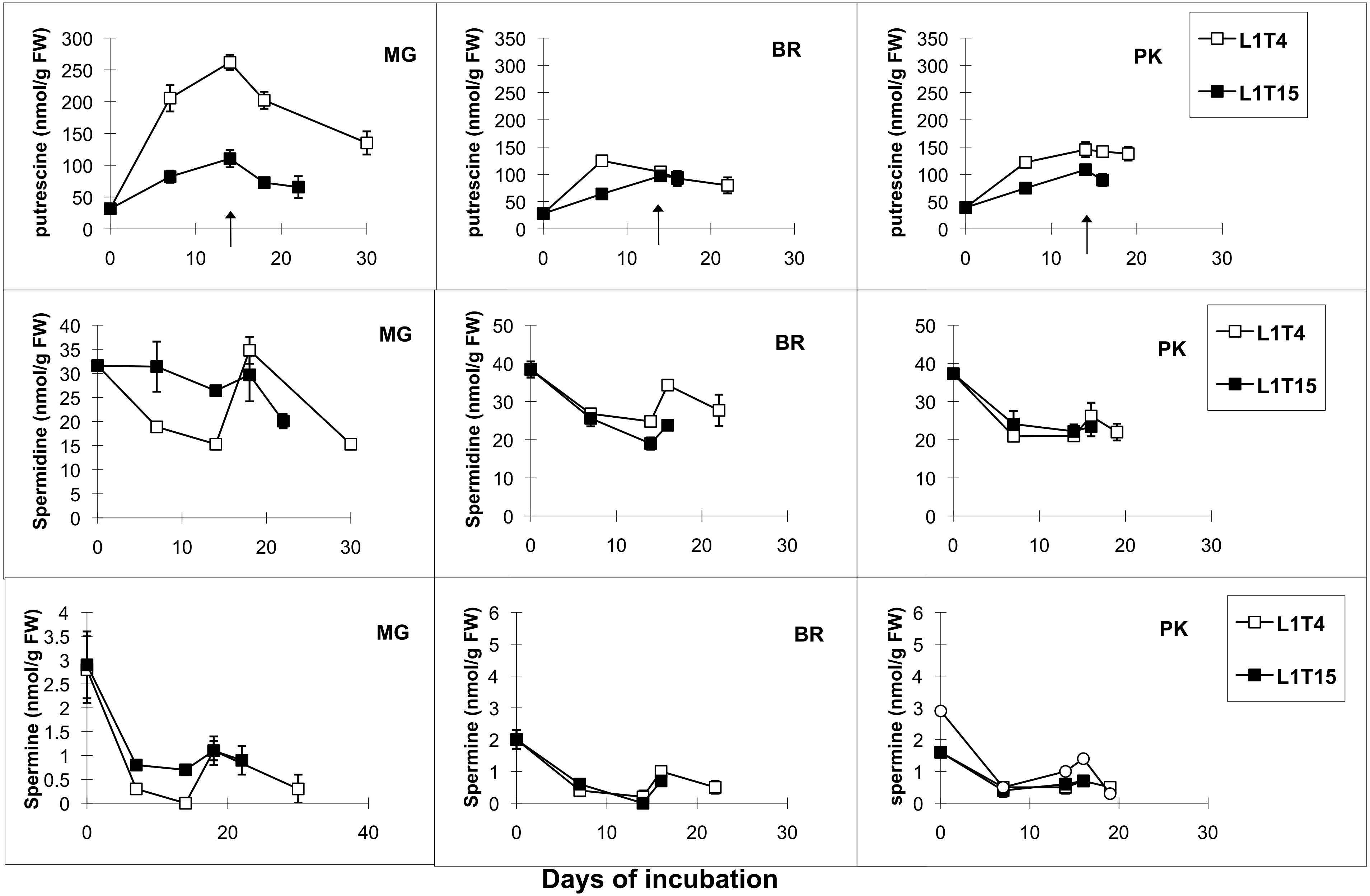

Supplementary Fig. 2. Profile of total soluble proteins as seen on Coomassie-blue R-250 stained SDS-PAGE gel in un-chilled and chilled and re-warmed fruits. The details of non-chilled and chilled-re-warmed fruits are same as described in Materials and Methods section.

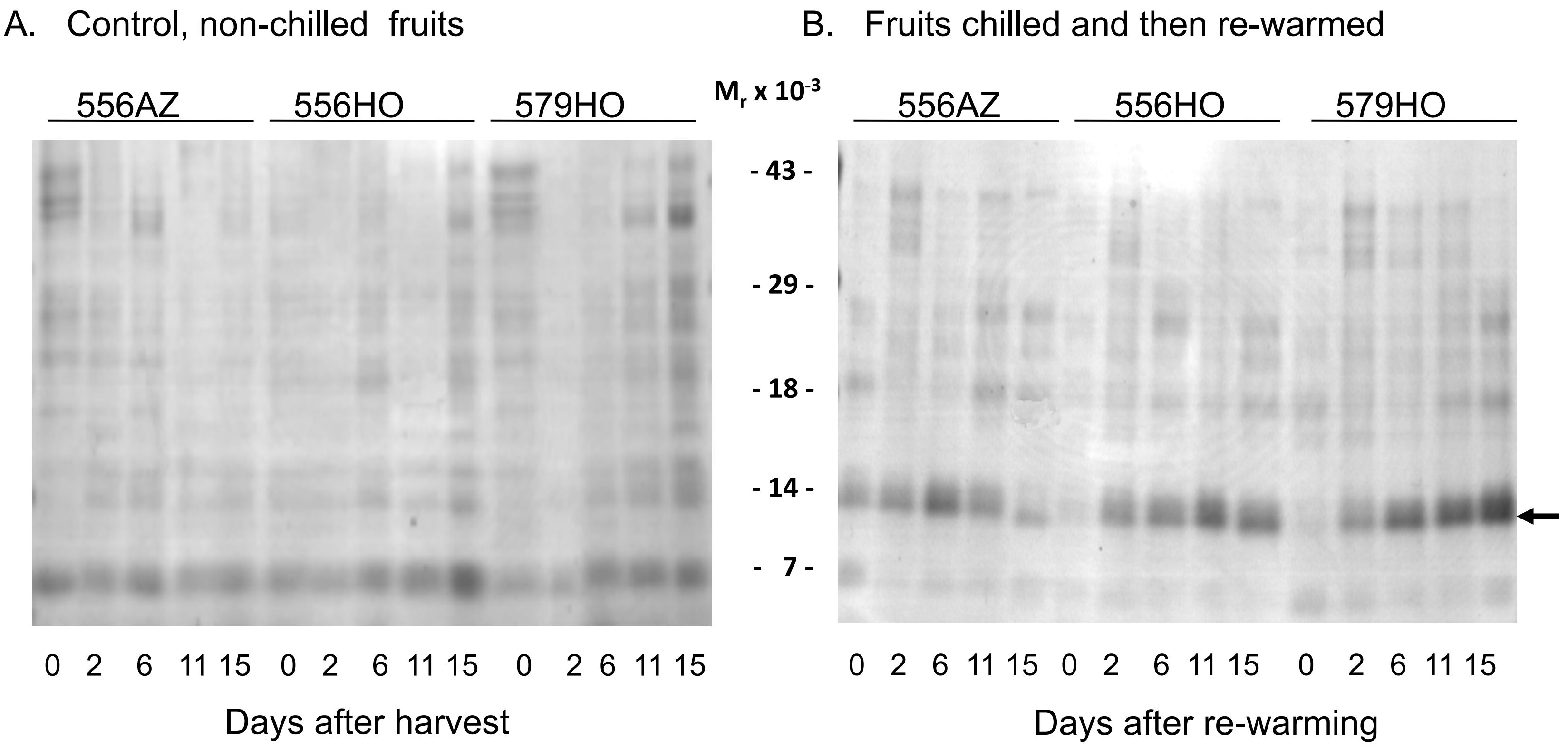

Supplement: Supplementary file 1 [file Data_Sheet_1.PDF]
